# The Impact of Fatigue on the Sense of Local and Global Rhythmic Movement

**DOI:** 10.5114/jhk/159604

**Published:** 2023-01-20

**Authors:** Michał Pawłowski, Mariusz P. Furmanek, Bogdan Bacik, Tomasz Skowronek

**Affiliations:** 1 Institute of Sport Sciences, The Jerzy Kukuczka Academy of Physical Education, Katowice, Poland.; 2 Department of Physical Therapy, University of Rhode Island, Kingston, RI, USA.; 3 Department of Physical Therapy, Movement and Rehabilitation Sciences, Northeastern University, Boston, USA.

**Keywords:** rhythm, coordination, jumping, frequency, variability, sex

## Abstract

Efficient movement control and the mechanisms responsible for the sense of rhythm are still not fully understood. The purpose of this paper was to estimate the influence of fatigue on the sense of rhythm defined as specific order of movements and their rhythmic perception. It was examined in a holistic way, by analyzing both global and local aspects of the movement. Twenty adult participants (20.2 ± 0.4 years, ten females) took part in the experiment. The fatigue protocol was applied in four blocks, which consisted of 30-s consecutive jumping with 80% of maximal effort. Immediately after each fatigue block, the rhythm performance was evaluated in global and local tests. The global test was based on 45 continuous jumps and was divided into an assisted and an unassisted phase using the Optojump Next System. The local test was performed by bilateral tapping of lower limbs by means of the Vienna Test System. The hypothesis about the significant effect of fatigue on the sense of rhythm was falsified. In particular, we observed the lack of differences between global and local aspects of the movement. Moreover, female participants showed a better sense of rhythm than males. Regardless of the fatigue protocol, participants made larger errors with a lower movement frequency in local rhythmic tasks. The coefficient of variation showed that sex differences were only significant in the unassisted phase of the global rhythmic task. We suggest that movement variability metrics may provide additional information about the sense of rhythm, which should be explored more in future studies, not only dependent on fatigue.

## Introduction

A sense of rhythm is crucial for efficient movement control. Generally, it is defined as specific order of movements and their rhythmic perception-motor skill responsible for producing the movements (Fraisse, 1984; Grahn, 2012). Having a sense of rhythm ensures timely movements of the limbs and joints, especially in cyclic motor tasks (Hogan and Sternad, 2007) which are involved in daily activities and intensive sports activities (MacPherson et al., 2009). Thus, rhythm should be considered a central part of effective preparation for sports performance. This is confirmed by numerous studies in sports disciplines and events like fencing (Hijazi, 2020), hurdles (Mehlich et al., 2002; Skowronek et al., 2013) and dancing (Minvielle-Moncla et al., 2008). Rhythm researchers agree on certain testing paradigms. Participants can perform rhythmic tasks in two ways: moving synchronously based on specific cues (an assisted rhythm/phase) or memorizing and reproducing the directed rhythmic pattern (an unassisted rhythm/phase). Both task variations can be perceived as local or global, depending on the number of muscles and joints involved in the movement. Most sense of rhythm studies are related to the local finger tapping task. Study populations vary from healthy adults (Reep, 2005), through musicians (Grahn and Rowe, 2009), patients (Konoike and Nakamura, 2020) and in specific groups of athletes (Mehlich et al., 2002). However, the finger-tapping test may not be an adequate method to evaluate the sense of rhythm in particular sports since the movement is specific to both the sports discipline and the environment in which it is played. Hence, the sense of rhythm in athletes has begun to be studied more globally in whole-body movements such as walking (Minvielle-Moncla et al., 2008), running (Skowronek et al., 2021), and jumping (Hijazi, 2020; Skowronek et al., 2013, 2018).

Sports performance in such specific conditions and at an increasing intensity of movement is undeniably linked to fatigue, which makes it one of the most principal factors in determining the training process and competition results (Enoka and Duchateau, 2016; Gates and Dingwell, 2008; Green, 1997). The intensity of the motions performed by athletes affects the physiological processes closely related to the nature of the currently performed movement (Enoka and Stuart, 1992; Enoka and Duchateau, 2008). The complex nature of fatigue in the performance of motor activities makes it difficult to be operationally defined. In this study, we define fatigue as a decrease in maximal force or power that the involved muscles can produce, and it develops gradually soon after the onset of the sustained physical activity (Enoka and Stuart, 1992; Enoka 1995; Enoka and Duchateau, 2008). Fatigue also could be a combination of central and peripheral body mechanisms and may disturb timing (Gandevia, 2001). For this reason, central (global) and peripheral (local) fatigue can be distinguished in the literature (Enoka, 1995). In the context of a sports performance and the sense of rhythm, the literature about fatigue is quite limited. To our knowledge, there are only few studies that have investigated local and global aspects of fatigue. For example, Waśkiewicz (2002) indicated the significant impact of fatigue after performing the Wingate test on the finger-tapping task. Lin et al. (2014) showed significant changes in rhythm performance in grip strength after a force production task and Hansen et al. (2014) showed no differences after fatiguing hip flexors and extensors in cycling tasks. However, as mentioned above, the method of local testing with finger tapping tasks seems inappropriate in sports performance. Thus, some authors have suggested using the global task. Hu and Ning (2015) measured the trunk motion at a standing position with a significant effect of fatigue. Other authors (Skowronek et al., 2018, 2021) also confirmed a significant influence of fatigue while running (under aerobic and anaerobic conditions), which impacted the sense of rhythm in a jumping test. It is also widely accepted that sex is a strong factor differentiating between the effects of fatigue (Côté, 2012; Paillard, 2012; Srinivasan et al., 2016). However, there is still much debate about the impact of sex on rhythmic performance, with several papers highlighting a lack of effect of sex on the sense of rhythm (Mehlich et al., 2002; Zachopoulou et al., 2000).

Despite all the literature mentioned above, the impact of fatigue on the sense of rhythm is still not fully understood. To our knowledge, no research has focused on the sense of rhythm holistically by simultaneously analyzing global and local features of the movement. Therefore, the main purpose of this study was to explore the effect of fatigue on the sense of rhythm in four ways: first, we tested the hypothesis (#1) that fatigue would have a significant impact on the sense of rhythm. Second, according to the novel approach, we hypothesized (#2) that the proposed fatigue protocol would impact a global, but not a local sense of rhythm. Third, we hypothesized (#3) that females would present a better sense of rhythm after fatiguing than males. Finally, we hypothesized (#4) that the phase of the rhythmic test (assisted vs. unassisted) would not be different between the sex of the participants in global rhythm.

## Methods

### Participants

Ten male (age: 20.22 ± 0.42 years, body mass: 74.11 ± 8.32 kg, and body height: 175.44 ± 4.91 cm; mean ± SD) and ten female adults (age: 20.2 ± 0.4 years, body mass 61.3 ± 7.86 kg, and body height: 165.5 ± 4.08 cm; mean ± SD) voluntarily participated in this study after signing an informed consent form approved by the Institutional Review Board of the Jerzy Kukuczka Academy of Physical Education in Katowice (7/2013). Participants had to meet the following inclusion criteria: (i) no history of any neurological or musculoskeletal disorder of lower limbs, (ii) a lack of experience of any regular sports training, and (iii) minimizing physical activity 48 hours before taking part in the experiment.

### Apparatus and the Experimental Procedure

**Figure 1:**
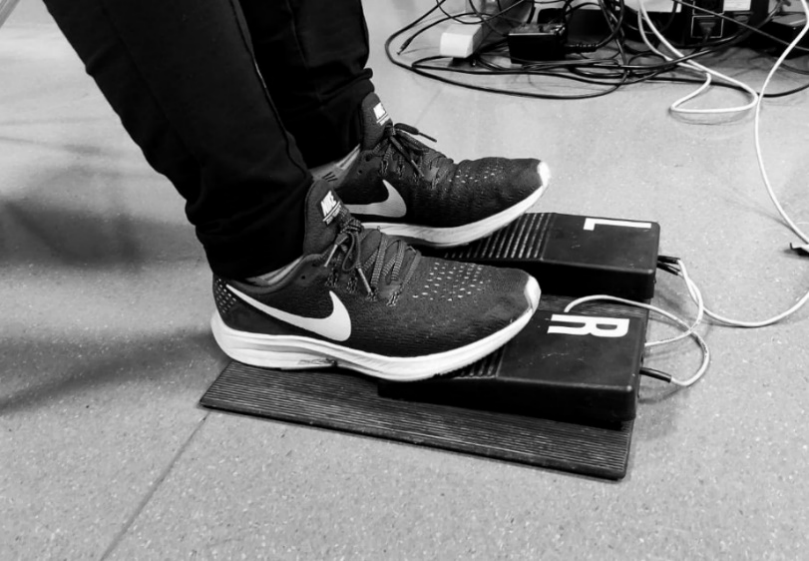
VST feet placement during the tapping test.

This study evaluated the effect of fatigue on the sense of rhythm in two ways: global and local. In order to assess a global rhythmic task, we used the Optojump Next system (Microgate, Italy), consisting of 100 diodes emitting infrared light, positioned 1 mm from the ground level at 10 mm intervals (diodes were built in long parallel bars (receiver and transmitter) lay on the flat floor). The Optojump Next system allowed to record data with a frequency of 1 kHz. The rhythm task consisted of 45 continuous jumps, where participants were instructed to jump rhythmically when an acoustic stimulus was provided by the metronome set at 1 Hz. The first 5 jumps of the test served to familiarize participants with the task and were not included in the analysis. The next 40 jumps were divided into two phases (20 jumps each): in the first phase (AP—assisted phase), participants jumped according to the metronome signal, whereas in the second phase (UAP— unassisted phase) participants jumped without any metronome signal. Participants were asked to remember the pace of the task during the first phase and continue it in the second phase without stopping. Participants’ hands were in the akimbo position (hands on hips with the elbows out wide) to eliminate the technique's influence on the jumping performance. The Optojump Next system is widely accepted as being both valid and reliable for vertical jumping (Słomka et al., 2017) and rhythmic motor tasks (Zając et al., 2012). Immediately after the first rhythmic task on the Optojump system, participants performed the second rhythmic task using the Vienna Test System (VST) (Schuhfried GMBH, Austria). In order to assess the local rhythmic task, we chose test number nine from the TAP-S1 parametric block (alternating the bilateral rhythm tapping test for lower limbs, which does not involve more than 30% of the body musculature). Participants were asked to sit comfortably and put their feet on the foot panel (peripheral panel of the VST) (Figure 1).

Participants were instructed to keep the rhythm produced by the VST (acoustic signal set at 0.833 Hz) by alternating pushing the assigned pedal on the mentioned panel. The local task consisted of the AP and UAP phases, each including 18 taps (9 for each foot). The reliability of the rhythmic test used was established in previous research (Raczek et al., 2001). The following variables of the collected data were analyzed: mean frequency of movements and mean absolute error (calculated as 1 minus observed frequency) for the UAP phase in both tasks, and mean frequency of movements, mean absolute error, and the coefficient of variance (CV) for both AP and UAP of the jumping task. All variables were normalized to the appropriate sampling rate of the rhythmic test.

**Figure 2:**
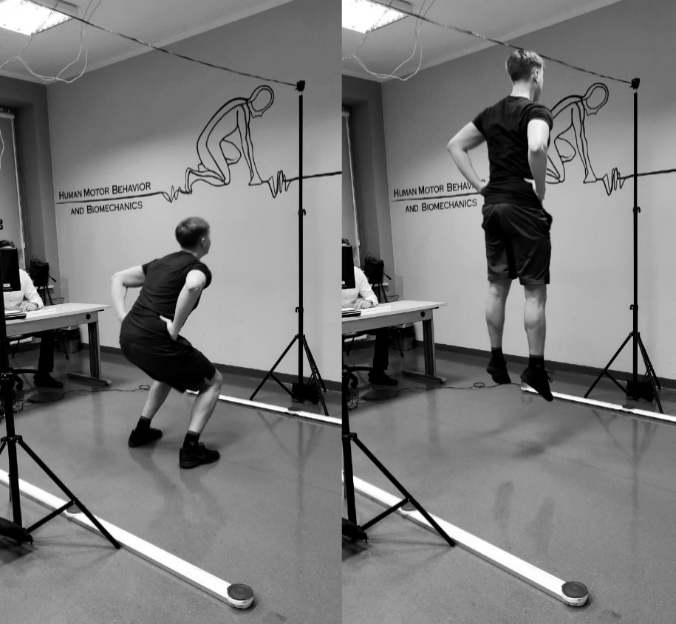
Placing of 80% of the maximal vertical jump target during the 30 s jumping test.

The data collection began from the execution of the described tests. In the next step, the fatigue protocol was started. Participants performed the 30-s jumping task (Dal Pupo et al., 2014) to the target height estimated at 80% of the maximal vertical jump height. According to recommendations (Bosco et al., 1983), participants were required to keep the trunk as vertical as possible, hands in the akimbo position and to flex their knees at 90° in the transition between the movement phases (eccentric-concentric). Verbal feedback was provided to the participants during the whole task and jump height was controlled. A line placed in the sagittal axis of the participant was used as a control target (Figure 2), which was to be achieved by the participant in every jump by touching it with the top of the head.

Immediately after finishing the 30-s jumping task (fatigue protocol), participants performed global (45 continuous jumps) and local (tapping) rhythmic tasks. This procedure was repeated for a total of five times, without a recovery time between the tasks.

### Statistical Analysis

Statistica 13 (StatSoft, USA) and Microsoft Excel 16 (Microsoft Inc., USA) were used for all analyses. Firstly, a 5×2×2 repeated measure analysis of variance (rm-ANOVA) including such factors as: *Fatigue* (Number of Repetition), *Type* of the task (Global, Local), and *Sex* (Female, Male) was used. Secondly, in order to compare the Phases in the global task, a 5×2×2 rm-ANOVA was also used with the following factors: *Fatigue* (Number of Repetition), *Phase* (Assisted, Unassisted), and *Sex* (Female, Male). Finally, a 5×2 rm-ANOVA was used to evaluate the impact of fatigue on the variability of the global task taking into account *Sex* only (Male, Female). The assumptions of the repeated measure ANOVAs were verified by the Shapiro-Wilk and Levene's tests. The Mauchly's test with the Greenhouse-Geisser corrections was used for violation of sphericity. Significant effects were further explored using pairwise contrasts with Bonferroni corrections. Each test statistic was considered significant at the alpha level of 0.05. All effect sizes are reported as partial eta-squared.

## Results

Neither movement frequency, nor movement error showed a significant main effect of *Fatigue* (*p* > 0.05), indicating that the fatigue protocol used in this study did not alter the sense of rhythm. Movement frequency showed a significant main effect of *Type* (*p* < 0.001) and *Sex* (*p* = 0.021), while movement error only showed a significant main effect of *Type* (*p* < 0.001). The analysis did not reveal any significant interactions between the main factors (Table 1).

**Table 1:** Outcomes of 5×2×2 rm-ANOVAs examining the impact of Fatigue, Type of the performed task (Global, Local), and Sex (Females, Males) on the sense of rhythm for analyzed variables.

Variable	Fatigue (F)	Type (T)	Sex (S)	Interactions
Hz (norm)	NS	F_(1,9)_ = 164.6	F_(1,9)_ = 7.78	NS
		*p*< 0.001	*p*= 0.021	
		*η^2^*= 0.948	*η^2^* = 0.464	
Error (norm)	NS	F_(1,9)_ = 251.484	NS	NS
		*p*< 0.001		
		*η^2^* = 0.965		

NS: not significant; η^2^: effect size explained with partial eta-square.

**Figure 3:**
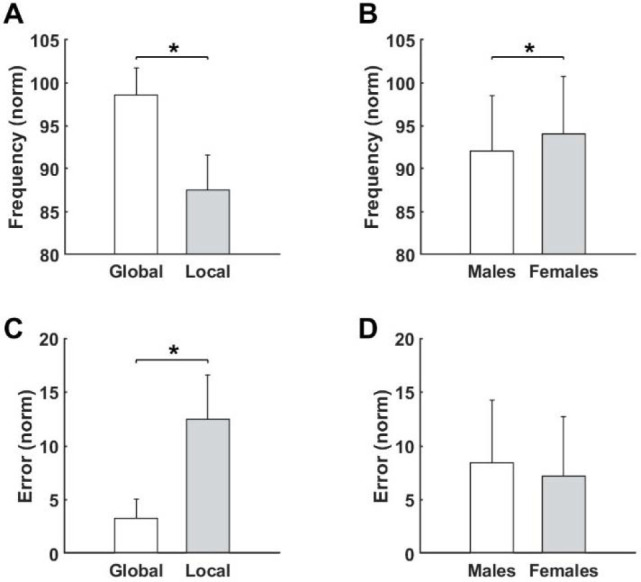
Differences for measured variables after fatigue for the Type of the performed task (A), (C) as well as for the Sex (B), (D). * significant difference (p < 0.05).

Interestingly, post-hoc analysis revealed that movement frequency was significantly larger in the global task than the local task (*p* < 0.001, Figure 3A). Moreover, the results showed that participants made more errors in local compared to global tasks (*p* < 0.001, Figure 3C). Furthermore, regardless of the task type, results showed that female participants achieved greater movement frequency than males (*p* = 0.021, Figure 3B). Movement error was not significant between the sexes (Figure 3D).

**Figure 4:**
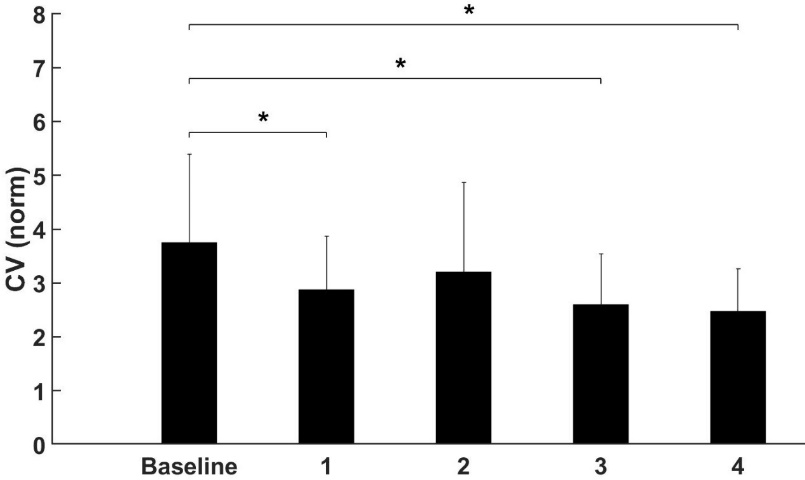
Significant effect of Fatigue on the CV in the global task. * significant difference (p < 0.05).

Similarly, to the former analysis, movement frequency and movement error did not show any significant changes after the fatigue protocol (*p* > 0.05, Table 2). However, a significant effect of fatigue on the variability of movement, expressed by the CV, was observed. The Bonferroni’s post hoc test revealed that the CV was significantly smaller after the first (*p* < 0.045), the third (*p* < 0.003) and the fourth (*p* < 0.001) repetition of the fatigue experiment when compared to the baseline measurement (Figure 4).

**Table 2:** Outcomes of 5×2×2 rm-ANOVAs examining the impact of Fatigue, Phase of the performed global task (Assisted, Unassisted) and Sex (Females, Males) on the sense of rhythm for analyzed variables.

Variable	Fatigue (F)	Phase (P)	Sex (S)	Interactions
Hz (norm)	NS	NS	F_(1,9)_ = 8.77	P*S
			*p*= 0.016	F(1,9) = 6.24
			*η^2^* = 0.494	*p*= 0.034
				*η2* = 0.409
Error (norm)	NS	F(1,9) = 7.615	F(1,9) = 11.028	NS
		*p*= 0.022	*p*= 0.009	
		*η2* = 0.458	*η2* = 0.551	
CV (norm)	F(2.08,18.73) = 6.379	F(1,9) = 9.5	NS	NS
	*p*= 0.007	*p*= 0.013		
	*η2* = 0.415	*η2* = 0.514		

NS: not significant; η^2^: effect size explained with partial eta-square.

**Figure 5:**
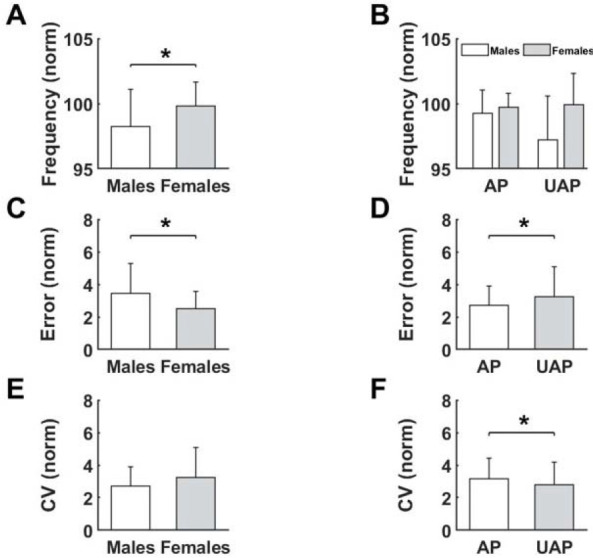
Differences for measured variables after Fatigue for the Sex (A, B, C, E) as well as for the Phase of the performed task (D), (F). * significant difference (p < 0.05).

Movement frequency showed a significant impact of *Sex*, and the interactions between the *Phase* of the global motor task and *Sex*. Females achieved greater values of movement frequency than males (*p* < 0.016, Figure 5A). We observed a decrease in movement frequency in males after changing the phase, yet an increase in females (*p* < 0.034, Figure 5B). The analysis performed for movement error confirmed earlier results. Again, females achieved lesser values of error than males (*p* < 0.009, Figure 5C). As expected, the analysis showed that movement error was higher in the UAP compared to the AP of the global test (*p* < 0.022, Figure 5D). The CV showed the opposite results than movement error. There were no significant differences between males and females (Figure 5E), and higher values were achieved in the AP than the UAP (*p* < 0.013, Figure 5F).

**Figure 6:**
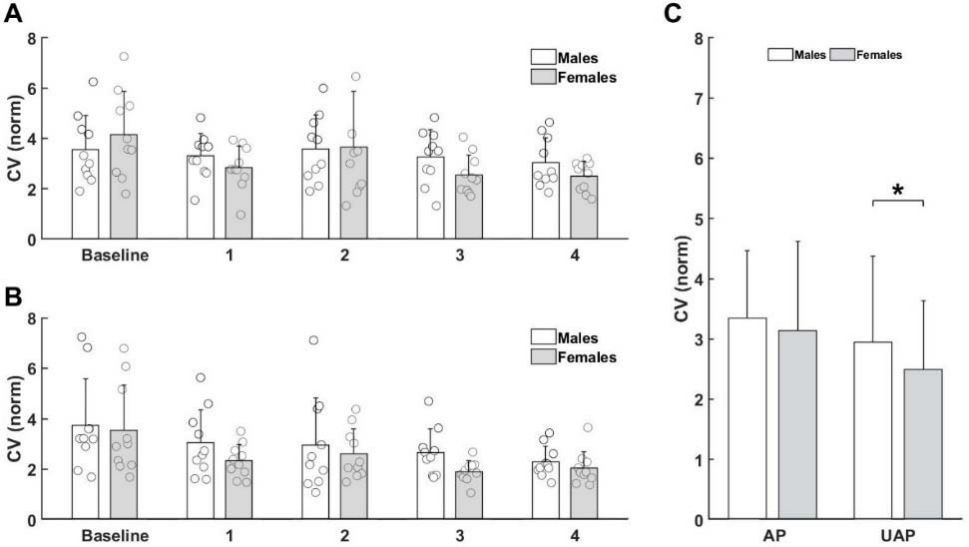
Significant main effect of Fatigue on the CV in the global task for the AP (A) and the UAP (B). Significant effect of Sex in the UAP (C). * significant difference (p < 0.05)

Finally, we investigated the effect of *Fatigue* and *Sex* differences on movement variability separately in the AP and the UAP of the global rhythmic task. The coefficient of variability showed a significant main effect of *Fatigue* for both the AP (Figure 6A) and the UAP (Figure 6B) (Table 3). Post-hoc tests revealed a decreased CV after four repetitions (*p* = 0.047) of the task in the AP and lesser values of the CV after the third (*p* = 0.011) and the fourth (*p* = 0.004) repetition of the task in the UAP. Importantly, the CV revealed *Sex* differences (F(1,9) = 4.632, *p* = 0.042) only during the UAP (Figure 6C). Male participants had a significantly higher CV (*p* = 0.042) than females.

**Table 3:** Outcomes of 5×2 rm-ANOVAs examining the impact of Fatigue and Sex (Females, Males) on the sense of rhythm for the CV in the global task.

Variable	Fatigue (F)	Sex (S)	Interactions
CV-AP	F_(4,36)_ = 3.374	NS	NS
	*p*= 0.019		
	*η^2^* = 0.273		
CV-UAP	F_(2.19,19.71)_ = 6.754	F_(1,9)_ = 5.644	NS
	*p*= 0.02	*p*= 0.042	
	*η^2^* = 0.34	*η^2^* = 0.385	

NS: not significant; η^2^: effect size explained with partial eta-square

## Discussion

The results showed that the proposed fatigue protocol induced by a repeated 30-s jumping test affected neither the global, nor the local sense of rhythm, which disproves hypotheses #1 and #2. Moreover, female participants showed a better sense of rhythm (higher movement frequency and lesser error) than males, thus confirming hypothesis #3. Interestingly, regardless of the fatigue protocol, we observed that participants made more errors with a lower frequency of movement in local rhythmic tasks. Additional analysis of the coefficient of variation showed that fatigue decreased movement variability in the global rhythmic task. Moreover, the coefficient of variation showed that sex differences were only significant in the unassisted phase of global rhythm. The observed decrease in variability and the increase in errors made within the unassisted phase of the rhythmic global task were mostly driven by the decreasing movement frequency of male participants. Males showed a greater coefficient of variation than females in the unassisted phase of the global rhythm, disapproving hypothesis #4.

## No Effect on the Global Rhythmic Task

The results of this investigation did not confirm our hypothesis (#2) that the proposed fatigue protocol would impact the global sense of rhythm. Insufficient intensity and relatively short time of the exercises might have explained that the structure of the global rhythm (frequency and error) was intact. However, when we analyzed global rhythm in the context of movement variability, we observed a decrease in the coefficient of variation after the completion of the fatigue protocol, suggesting compensation in motor strategies. For instance, paraspinal muscles might show a greater frequency of anticipatory involvement than leg muscles (Strang et al., 2008) and/or cognitive resources may be increased (Paillard, 2012). The last factor was evident in the unassisted phase of the test, where a high vigilance level was crucial to maintain the temporal structure (Nardone et al., 1998).

The results of this investigation contradict our previous study (Skowronek et al., 2021), where fatigue was induced by two running-based sprint anaerobic tests (RASTs), which most likely induced greater fatigue. We believe that there are few factors that could cause a lack of changes in the global rhythmic task after the fatigue protocol. The first factor to be considered was described by Gutin (1973). Results of that study showed that different kinds of fatigue at the same intensity level, but induced by different movement patterns (running, jumping, cycling), might affect the sensorimotor and cognitive abilities differently. It is possible that global movements, such as jumping with hands on the hips, may be considered less “global” than sprinting due to different complexities of mechanical and sensorimotor coordination needed for such movements. Therefore, fatigue induced by jumping may have led to less control of the rhythm. This hypothesis is supported by Bonnard and colleagues’ (1994) research. In their experiment, just as in ours, participants were asked to jump to induce general (global) fatigue. They performed two-leg jumps with their arms crossed on the chest for as long as possible. The time of this experiment varied from 23 to 44 min of continuous jumping. The intensity (height of jump range) was set individually, but the frequency was held at 2 Hz for all participants; however, keeping the pace was not the experiment's aim. The authors concluded that even after a long, exhausting protocol, sensorimotor coordination and the movement pattern were not disturbed. The second factor may be related to the compensatory mechanisms (Rodacki et al., 2002), which reduce the fatigue consequences. Other studies involving running, sprinting (Goodall et al., 2015) and continuous hopping exercises (Dal Pupo et al., 2013) provide evidence that some compensatory mechanisms are used to counterbalance the loss of the muscle force-generating properties caused by fatigue. In our experiment, the fatigue protocol and the global rhythm test were based on the same movement pattern, i.e., jumping, which undoubtedly gave an advantage to participants and might have affected the results of the rhythm test. The pace of the test was imposed by a metronome for one of the phases, which helped participants with movement optimization, resulting in lower effort during the test execution (MacPherson et al., 2009).

## No Effect on the Local Rhythmic Task

In the local rhythmic task, both feet were alternately tapping, hence we did anticipate that no effect would be observed (hypothesis #2). We did not measure the lactate (LA) concentration during the experiment, but instead relied on previous research by Beneke et al. (2004) and our previous study (Skowronek et al., 2021) to make the assumption that upon the last repetition of the global fatigue protocol, LA was above 12 mmol/l. However, the proposed global fatigue protocol did not trigger the central mechanism of fatigue and the local effects of it were probably neutralized by the mechanism mentioned above.

Another explanation of the lack of the effect of fatigue on local rhythm may be related to the theory about the existence of preferred coordination patterns in the human nervous system (Swinnen and Wenderoth, 2004). For instance, bimanual finger taping tasks in phase movement patterns are most stable at high movement frequencies (similar muscles). In contrast, anti-phase movement patterns (non-similar muscles) can be maintained at low movement frequency only. The simple movement pattern (1:1) of alternating bilateral feet tapping required low frequency (< 1 Hz) and the activation of homologous muscles allowed for robust performance after the global fatigue protocol.

### Woman Showed a Better Sense of Rhythm

There is evidence that women are usually less fatigable than men for the similar intensity of exercises, as confirmed by Hunter (2014). This may explain why we observed a better sense of rhythm in female than male participants after the fatigue protocol. Hunter (2009, 2018, 2014) also described a number of physiological mechanisms responsible for sex differences in fatigue, including activation of the motor neuron pool from cortical and subcortical regions, synaptic inputs to the motor neuron pool via activation of metabolically-sensitive small afferent fibers in the muscle, muscle perfusion, skeletal muscle metabolism, fiber type properties, and pain management. Females showed a higher frequency of the rhythmic task and were able to keep this during the unassisted phase, suggesting superior cognitive and executive functions than male participants.

## Conclusions

As the literature shows (Noakes, 2007), fatigue results from complex peripheral physiological system and brain interactions. We assume that these interaction mechanisms could be involved in preventing excessive peripheral and central fatigue. This could be the cause of no effect of the global fatigue protocol on either global or local senses of rhythm. Despite the study's limitations listed below, it is possible that the rhythmic coordination modes are sustained and robust to fatigue, particularly during low-frequency tests. We also confirmed that female participants presented a superior sense of rhythm. Movement variability metrics may provide additional information about the sense of rhythm and thus, should be explored more in future rhythm studies.

## Limitations

The study has several limitations which should be considered in follow-up studies. One of them is the lack of objective physiological measurements that indicate the baseline level of fatigue (e.g., percentage of the max heart rate, LA) and subjective data related to participants’ fatigue (e.g., VAS, Borg scales). The results suggest that the intensity of 80% maximal jump height could not be sufficient to evaluate the fatigue phenomenon. Another limitation is the lack of randomization of measures. The local rhythmic task could be performed when the effects of fatigue have vanished. However, the time between the fatigue inducing exercise and the local rhythmic task was shorter than three minutes, and participants could not fully recover. The last limitation of this study is that there was no entirely ecological comparison between local-global rhythm tests due to a difference between global and local tasks. Future studies are needed to ensure coherence within the used motor tasks.
